# Stratification of follicular thyroid tumours using data‐independent acquisition proteomics and a comprehensive thyroid tissue spectral library

**DOI:** 10.1002/1878-0261.13198

**Published:** 2022-03-12

**Authors:** Yaoting Sun, Lu Li, Yan Zhou, Weigang Ge, He Wang, Runxin Wu, Wei Liu, Hao Chen, Qi Xiao, Xue Cai, Zhen Dong, Fangfei Zhang, Junhong Xiao, Guangzhi Wang, Yi He, Jinlong Gao, Oi Lian Kon, Narayanan Gopalakrishna Iyer, Haixia Guan, Xiaodong Teng, Yi Zhu, Yongfu Zhao, Tiannan Guo

**Affiliations:** ^1^ 12377 Zhejiang University Hangzhou China; ^2^ Westlake Laboratory of Life Sciences and Biomedicine Key Laboratory of Structural Biology of Zhejiang Province School of Life Sciences Westlake University Hangzhou China; ^3^ Institute of Basic Medical Sciences Westlake Institute for Advanced Study Hangzhou Zhejiang China; ^4^ Westlake Omics (Hangzhou) Biotechnology Co., Ltd Hangzhou China; ^5^ Whiting School of Engineering Department of Biomedical Engineering Johns Hopkins University Baltimore MD USA; ^6^ Department of General Surgery The Second Hospital of Dalian Medical University Dalian China; ^7^ Department of Urology The Second Hospital of Dalian Medical University Dalian China; ^8^ Division of Medical Sciences National Cancer Centre Singapore Republic of Singapore; ^9^ Department of Head and Neck Surgery National Cancer Centre Singapore Republic of Singapore; ^10^ Department of Endocrinology Guangdong Provincial People's Hospital Guangdong Academy of Medical Sciences Guangzhou China; ^11^ Department of Pathology The First Affiliated Hospital Zhejiang University School of Medicine Hangzhou China

**Keywords:** data‐independent acquisition, mass spectrometry, proteomics, spectral library, thyroid nodules

## Abstract

Thyroid nodules occur in about 60% of the population. A major challenge in thyroid nodule diagnosis is to distinguish between follicular adenoma (FA) and carcinoma (FTC). Here, we present a comprehensive thyroid spectral library covering five types of thyroid tissues. This library includes 121 960 peptides and 9941 protein groups. This spectral library can be used to quantify up to 7863 proteins from thyroid tissues, and can also be used to develop parallel reaction monitoring (PRM) assays for targeted protein quantification. Next, to stratify follicular thyroid tumours, we compared the proteomes of 24 FA and 22 FTC samples, and identified 204 differentially expressed proteins (DEPs). Our data suggest altered ferroptosis pathways in malignant follicular carcinoma. In all, 31 selected proteins effectively distinguished follicular tumours. Of those DEPs, nine proteins were further verified by PRM in an independent cohort of 18 FA and 19 FTC. Together, we present a comprehensive spectral library for DIA and targeted proteomics analysis of thyroid tissue specimens, and identified nine proteins that could potentially distinguish FA and FTC.

## Graphical abstract

Here we present a comprehensive spectral library of 9941 protein groups for investigating the proteome of thyroid tissues. By using the spectral library and data‐independent acquisition (DIA) mass spectrometry, we identified nine differentially expressed proteins that could distinguish follicular thyroid adenoma from follicular thyroid carcinoma.

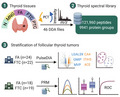

**Abbreviations**
AGC: automatic gain control
CA4: carbonic anhydrase 4
CV: coefficient of variation
DEP: differentially expressed protein
DDA: data‐dependent acquisition
DIA: data‐independent acquisition
FA: follicular adenoma
FDR: false discovery rate
FFPE: formalin‐fixed paraffin‐embedded
FTC: follicular thyroid carcinoma
FWHM: full widths at half maximum
GMIP: GEM‐interacting protein
GO: gene ontology
IAA: iodoacetamide
IPA: ingenuity pathway analysis
ITIH5: inter‐alpha‐trypsin inhibitor heavy chain H5
LC: liquid chromatography
LGALS9: galectin‐9
LRP4: LDL‐receptor‐related protein 4
MNG: multinodular goitre
MS: mass spectrometry
MVP: major vault protein
PCT: pressure cycling technology
PRM: parallel reaction monitoring
PTC: papillary thyroid carcinoma
PTPRE: protein tyrosine phosphatase receptor type E
qPCR: quantitative polymerase chain reaction
SMOC2: SPARC‐related modular calcium binding 2
SVM: support vector machine
TCGA: The Cancer Genome Atlas
TFA: trifluoroacetic acid
TMA: tissue microarrays
t‐SNE: t‐distributed stochastic neighbour embedding

## 1 Introduction

Thyroid nodules are common and, given the sensitivity of current diagnostic techniques, can be detected in approximately 60% of the general population, especially in women [1, 2]. The incidence of thyroid malignancy or thyroid carcinoma has rapidly increased over the last decades, although it is uncertain if this is a real increase or simply a result of widespread use of screening ultrasonography [3, 4]. Most of these nodules are asymptomatic. Only 4–7% of patients present with complaints attributed to thyroid nodules. Although ultrasonography and ultrasound‐guided fine‐needle aspiration can help distinguish between benign and malignant nodules, approximately 30% of thyroid nodules remain indeterminate by cytopathology and require diagnostic surgery [5], after which histopathology of surgical specimens provides a definitive and complete diagnosis. Only about 15% of indeterminate nodules are proved to be malignant. Because many benign nodules are clinically ambiguous and a source of uncertainty, such patients often undergo unnecessary surgery. Most ambiguous diagnosis occurs in follicular tumours, which constitute about 30% of the indeterminant nodules [6]. The benign follicular tumours (i.e. follicular adenoma, FA) could not be separated from the malignant nodules (i.e. follicular thyroid carcinoma, FTC) by cytologic, sonographic or clinical features [7]. The only way to separate them is to perform a diagnostic surgery, and examine tumour cell invasion under microscopy. Therefore, there is an urgent need to identify molecular markers to distinguish them. Several nucleic‐acid‐based molecular tests based on next‐generation sequencing technology [8] have been developed for the diagnosis of indeterminant thyroid nodules; however, no genomic and transcriptomic signature has been identified to distinguish FA and FTC.

Unlike nucleic acids, proteins are directly involved in all life processes and determine cellular and organismal phenotypes. Proteins can be effective diagnostic biomarkers and therapeutic targets. Mass spectrometry (MS)‐based proteomics has reached a high level of technical and methodological development during the last decade. Data‐independent acquisition (DIA), in particular, enables comprehensive quantitation of peptides from complex compositions with high reproducibility and throughput [9]. In the conventional data‐dependent acquisition (DDA) mode, only peptide precursors with high abundance in MS1 are fragmented. In DIA, however, all flyable peptide precursors within a predefined range (also called window) of mass‐to‐charge ratio (*m/z*) are fragmented by sequential repetitive cycling in windows, thus providing detailed data without loss of any eluted peptides [9, 10]. The pressure cycling technology (PCT)‐based sample preparation methodology, coupled with DIA‐MS, allows proteomic analysis of biopsy‐level tissues within 6 h [11, 12].

To optimize the accuracy of spectral identifications, DIA data analysis requires tissue‐ or organism‐specific spectral libraries [10, 13]. Although pan‐human libraries derived from multiple human species and cell lines have been reported [14, 15], these comprehensive libraries could cause inaccuracies during ion matching, which can be partly alleviated by subLib strategy [16]. In recent years, several novel software for DIA data analysis, such as DIA‐Umpire [17], PECAN [18] or DIA‐NN [19], no longer require spectral libraries. However, this library‐free mode should be applied with caution because of its relatively lower protein identification power compared to a library‐based strategy, particularly for small DIA data sets [20]. A tissue‐specific library for thyroid nodules, both benign and malignant, as well as for healthy thyroid, would provide a useful resource for proteomic analysis of thyroid tissues in a high‐throughput manner.

## 2 Materials and methods

### 2.1 Sample collection

For the spectral library construction, validation and application, normal thyroid tissue and thyroid nodular samples from patients aged 18 years or older were collected, between 2011 and 2019, from three clinical centres in Singapore (Singapore General Hospital) and China (The Second Hospital of Dalian Medical University and the First Affiliated Hospital of Zhejiang University). Tissue cores of 1 mm diameter (0.6–1.2 mg) were extracted from the pathological regions of interest in formalin‐fixed paraffin‐embedded (FFPE) tissue blocks demarcated by experienced histopathologists [21].

To establish a comprehensive thyroid‐specific spectral library, we included four common histopathological types of thyroid nodules (42 multinodular goitre (MNG), 49 FA, 33 FTC and 54 papillary thyroid carcinoma (PTC)), as well as 10 normal thyroid tissues (N). To further validate our library, three PTC samples, together with paired pericancer tissues were collected from the Second Hospital of Dalian Medical University. We also separately assembled two histopathological types of follicular thyroid tumours, FA and FTC, from two clinical centres as proof of principle for clinical diagnostic application. Twenty‐four FA and 22 FTC were collected from the Second Hospital of Dalian Medical University, and the other 18 FA and 19 FTC were from the First Affiliated Hospital of Zhejiang University. Detailed patient characteristics are listed in Table S1. Ethics approval was obtained from all the three hospitals and Westlake University. The experiments were undertaken with the understanding and written consent of each subject, and according to Helsiki declaration.

### 2.2 Sample preparation assisted by PCT

The tissue samples were prepared for proteomic analysis as described previously [11, 22]. Samples were dewaxed, hydrated and acidified using heptane, a decreasing ethanol series (100%, 90% and 75%), and 0.1% formic acid in sequence. The samples were next kept under basic hydrolysis conditions in Tris‐HCl (100 mm, pH = 10) at 95 °C for 30 min and then transferred to a solution containing 30 µL lysis buffer (6 m urea, 2 m thiourea), 5 µL Tris (2‐carboxyethyl) phosphine (TECP, 10 mm) and 2.5 µL iodoacetamide (IAA) (40 mm). In PCT‐Micro Tubes, samples were lysed, reduced and hydroxylated at 30 °C using PCT (90 cycles, 45 000 psi, 30 s on‐time and 10 s off‐time). Trypsin (enzyme‐to‐substrate ratio, 1:50; Hualishi Scientific, China) and LysC (enzyme‐to‐substrate ratio, 1:40; Hualishi Scientific, China) were then added, followed by PCT‐assisted digestion (120 cycles, 20 000 psi, 50 s on‐time and 10 s off‐time). 1% trifluoroacetic acid (TFA) was added to terminate the digestion process. The resulting peptides were desalted with 2% acetonitrile (ACN) and 0.1% TFA and reconstituted. Peptide concentrations were measured with a Nanoscan (Analytic Jena, Germany) at A_280_, and samples were stored at 4 °C for further analysis. All the chemical reagents, unless specified, were obtained from Sigma‐Aldrich.

### 2.3 Strong cation exchange (SCX) fractionation of peptides

Clean peptides were fractionated by 100 mg SCX solid‐phase extraction (SPE) columns (HyperSep™, Thermo Fisher Scientific, San Jose, CA, USA) to enhance the peptide coverage. 600 µg of pooled peptides, including all five types of thyroid tissues (10 N, 42 MNG, 28 FA, 13 FTC, 38 PTC), was reconstituted in equilibration buffer (2.5 mm KH_2_PO_4_/25% ACN, pH = 3.0). SCX columns were washed with ddH_2_O water and equilibration buffer. The pooled sample was then loaded onto conditioned cartridges. Loaded columns were washed with six diluents with different ratios of buffer A (10 mm KH_2_PO_4_/25% ACN, pH = 3.0) to buffer B (10 mm KH_2_PO_4_/1 m KCl/25% ACN, pH = 3.0) to introduce increasing KCl concentrations. The samples were then split into six fractions for each sample and cleaned by C18 spin columns (The Nest Group, USA).

### 2.4 High‐pH reversed‐phase chromatography fractionation of peptides

To further increase the peptide coverage in the spectral library, another fractionation method, that is, high‐pH reversed‐phase chromatography, was performed. Two pooled samples were combined from 41 follicular thyroid neoplasms (21 FA and 20 FTC) and 16 PTC samples. ~ 200 μg of each pooled sample was separated by Thermo Dinex Ultramate 3000 with an XBridge peptide BEH C18 column (4.6 mm × 250 mm, 5 μm, 1·pkg^−1^) at 45 °C. The gradient was 60 min long, with a flow rate of 1 mL·min^−1^, and the mobile phase consisted of buffer A (ddH_2_O water with 0.6% ammonia, pH = 10) and buffer B (98% ACN with 0.6% ammonia, pH= 10). The gradient was from 5% to 35% buffer B in a condition of pH 10.0 at a flow rate of 1 mL·min^−1^. In all, 60 fractions were collected at 1 min separation interval. The 60 fractions were subsequently combined into 20 fractions for each pooled sample to build the spectral library. The resulting fractionated peptides were then resuspended into 20 µL buffer (2% of ACN, 0.1% FA) for MS injections.

### 2.5 DDA

The fractionated peptides spiked with iRT (Biognosys, Schlieren, CH) at a concentration of 10% were separated by UltiMate™ 3000 RSLCnano System (Thermo Fisher Scientific). The system was equipped with a homemade 15 cm × 75 µm silica column custom packed with 1.9 µm 100 Å C18‐Aqua. The mobile phase comprised buffer A (2% ACN, 0.1% formic acid) and buffer B (98% ACN, 0.1% formic acid). Peptides were separated on a 60 min effective liquid chromatography (LC) buffer B gradient (3% to 28% at 300 nL·min^−1^). Ionized peptides were transferred into a Q Exactive^TM^ HF MS (Thermo Fisher Scientific). Full MS scans were measured with an Orbitrap at a resolution of 60 000 full widths at half maximum (FWHM) at *m/z* of 200 Th covering 400–1200 Th precursors, with automatic gain control (AGC) target value of 3E6 charges and 80 ms maximum injection time (max IT). The top 20 precursor signals were chosen to be fragmented in a higher energy collisional dissociation cell with 27% normalized collision energy and then transferred to an Orbitrap for MS/MS analysis at a resolution of 30 000 FWHM and an AGC target value of 1E5. Using 60 min LC gradients, we acquired 46 DDA files. More details are listed in Table S2.

### 2.6 Spectral library construction

Spectronaut™ Pulsar X version 14.6 (Biognosys) was used to generate a spectral library specific to the thyroid tissues. All 46 DDA raw files were searched by Pulsar against a human Swiss‐Prot FASTA database (downloaded on 2020‐01‐22) which included 20 367 protein sequences with false discovery rate (FDR) of 0.01. The enzyme was set to ‘trypsin/P’ allowing no more than two missed cleavages; cysteine carbamidomethyl was set to a fixed modification, while methionine oxidation was set to a variable modification; mass tolerance was automatically determined, while other settings were default.

### 2.7 Quantitative analysis of thyroid samples by DIA and PulseDIA

Together with paired pericancer tissues, three PTC samples were prepared as previously described [11, 22]. Proteomic data for these tested thyroid samples were acquired by DIA or PulseDIA, a gas phase fractionation method [23]. The LC effective gradient was 45 min for each run, with 3%–25% buffer B at 300 nL·min^−1^. MS1 was performed over an *m/z* range of 390–1010 Th for the DIA, and 390–1210 Th for the PulseDIA, with a resolution of 60 000 FWHM, an AGC target of 3E6 and a max IT of 80 ms. MS2 was performed with a resolution of 30 000 FWHM, an AGC target of 1E6 and a max IT of 55 ms. For DIA, 24 isolation windows were performed: 20 with *m/z* of 21 Th windows, 2 with 41 Th windows and 2 with 61 Th windows. For PulseDIA, five or four injections with 24 isolation windows per injection were performed [23]. DIA data were analysed by Spectronaut™ (version 14.6) and DIA‐NN (version 1.7.15). All settings were set to their default values.

### 2.8 Targeted proteins analysis by parallel reaction monitoring (PRM)

PRM validation was performed on 37 selected proteins. Six proteins were chosen from differentially expressed proteins (DEPs) with adjusted *P* value less than 0.05. And the other 31 proteins were originated from geNetClassifier selection. We selected at least one peptide precursor for one protein to be monitored with the limitation of no modification and missed cleavage and the peptide length ranging from 8 to 20 using skyline (Version 21.1). PRM was performed on a Q Exactive^TM^ HF MS (Thermo Fisher Scientific) system with UltiMate™ 3000 RSLCnano System (Thermo Fisher Scientific). Cleaned peptides from FA and FTC samples were separated at a flow rate of 300 nL·min^−1^ along a 60 min 10%–30% linear LC buffer B effective gradient. The mobile phase buffers were the same as the buffers mentioned in the method of DDA section. The time‐scheduled acquisition was in a ± 3 min retention time window. The full scans were collected with *m/z* from 300 to 2000 Th with a resolution of 60 000 FWHM. The AGC target was 3E6 and the maximum injection time was 55 ms. Target precursors were then isolated through an *m/z* window of 1.6 Th, followed by fragmentation at 27% normalized collision energy. The product ions were scanned with a resolution of 30 000 FWHM, AGC target value of 2E5 charges and maximum injection time of 100 ms. Here, 26 peptide precursors from 22 proteins and 20 CiRT peptide precursors were analysed.

### 2.9 Classification of FTC and FA

To distinguish FTC from FA, two data matrices were acquired. The PulseDIA matrix was produced from 24 FA and 22 FTC samples (the Second Hospital of Dalian Medical University), and the PRM matrix was acquired from 18 FA and 19 FTC samples (the First Affiliated Hospital of Zhejiang University). The proteomic data matrices were log_2_ transformed, and then the missing values for each protein were imputed by 0.8* minimum of their non‐missing values.

R package geNetClassifier provides a method to identify a minimum subset within the input protein set that can be used to distinguish disease subtypes [24]. In brief, the package uses an unsupervised way (parametric empirical Bayes) first to rank the input proteins and keep only the proteins whose posterior probabilities are above the threshold (95%) as candidate features to build a support vector machine (SVM) classifier to stratify thyroid nodules. Global accuracy and call rate were calculated to evaluate the performance of the SVM classifier. Default parameters were set for the protein selection and classification by geNetClassifier in this study. A subset of proteins within the input protein set, that is, PulseDIA matrix of 204 differentially expressed proteins (DEPs), were selected to build up a linear SVM classifier.

Among the 37 proteins (six proteins from DEPs and 31 proteins from geNetClassifier) detected by PRM, nine proteins were confirmed to be significantly dysregulated (adjusted *P* value < 0.05) in an independent sample set. The PRM dataset was randomly divided into training (*n* = 25) and testing (*n* = 12) sets. Based on R package mlr3 [25], we built an SVM model on the training set (*n* = 25) with the nine proteins using the same hyper‐parameters setting (linear kernel, unit cost of constraints violation) as previously employed in 31 proteins of PulseDIA data and evaluated the model on the testing set (*n* = 12).

### 2.10 Statistical and bioinformatic analyses

Statistical analysis was performed using r software (version 3.5.1). Protein annotation was performed by Ingenuity Pathway Analysis (IPA) and the database of KinMap [26]. DEPs were analysed by the R package of limma with a threshold of *P* value less than 0.05 and BH adjusted *P* value less than 0.05. Heatmap was drawn by R package of pheatmap. Hierarchical clustering after row normalization with default parameter setting in pheatmap. Pathway enrichment was performed by IPA, in which *P* value was calculated by right‐tailed Fisher’s exact test. Statistical significance in the box plots was calculated by two‐tailed Student’s *t*‐test.

## 3 Results

### 3.1 Thyroid spectral library construction

**Fig. 1:**
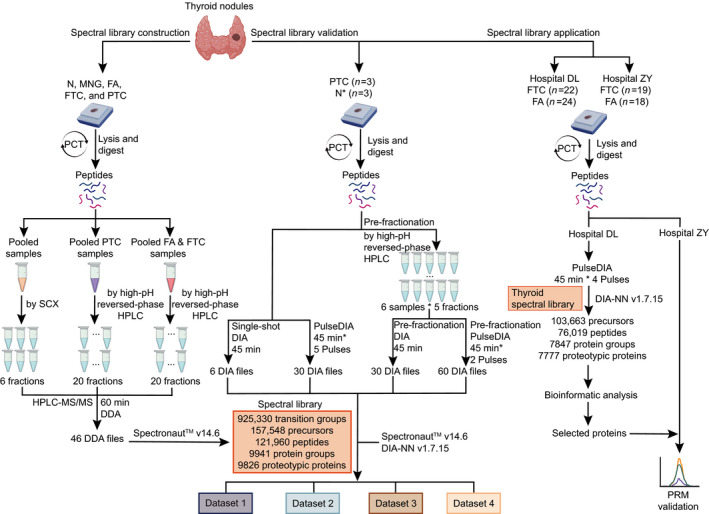
Generation, validation and application of a comprehensive thyroid‐specific spectral library. Left panel, generation of the spectral library. Five types of thyroid tissues were collected and prepared for proteomic analysis using pressure cycling technology (PCT). Three pooled thyroid samples were fractionated using strong cation exchange (SCX) or high‐pH reversed‐phase chromatography. Each peptide fraction was analysed using data‐dependent acquisition (DDA) MS for spectral library generation using Spectronaut v14.6. Middle panel, validation of the spectral library. The established library was validated by four DIA data acquisition strategies. Right panel, application of spectral library for proteomic analysis of follicular thyroid adenoma and carcinoma in a multicentre study. N*, the pericancer tissues.

This study introduces a thyroid‐specific spectral library to support protein identification and quantification in thyroid nodules by DIA‐MS (Fig. 1). Five types of thyroid tissues were collected, namely normal tissues, two types of benign nodules MNG and FA, and two types of thyroid carcinomas FTC and PTC. Normal thyroid and thyroid nodule tissues were processed by PCT; extracted and desalted peptides were then combined into three different pooled samples: (a) pooled sample containing all five types, (b) PTC pooled sample, and (c) FA and FTC pooled sample. The pooled peptides were fractionated in two ways, that is, SCX or high‐pH reversed‐phase chromatography, to achieve higher peptide coverage. Peptide fractions were injected into HPLC‐MS/MS with 60 min‐gradient using DDA‐MS. We totally acquired 46 DDA files. Our thyroid‐specific spectral library comprises 925 330 transition groups, 157 548 precursors, 121 960 peptides, 9941 protein groups and 9826 proteins from proteotypic peptides (Table 1). We then validated this library by four DIA datasets acquired with four different acquisition strategies and applied it in proteomic stratification of FA and FTC (Fig. 1).

**Table 1:** Statistics of the thyroid‐specific spectral library

	Library
Transition groups	925 330
Peptide precursors	157 548
Peptides	121 960
Protein groups	9941
Proteotypic proteins	9826

### 3.2 Characteristics of thyroid spectral library

**Fig. 2:**
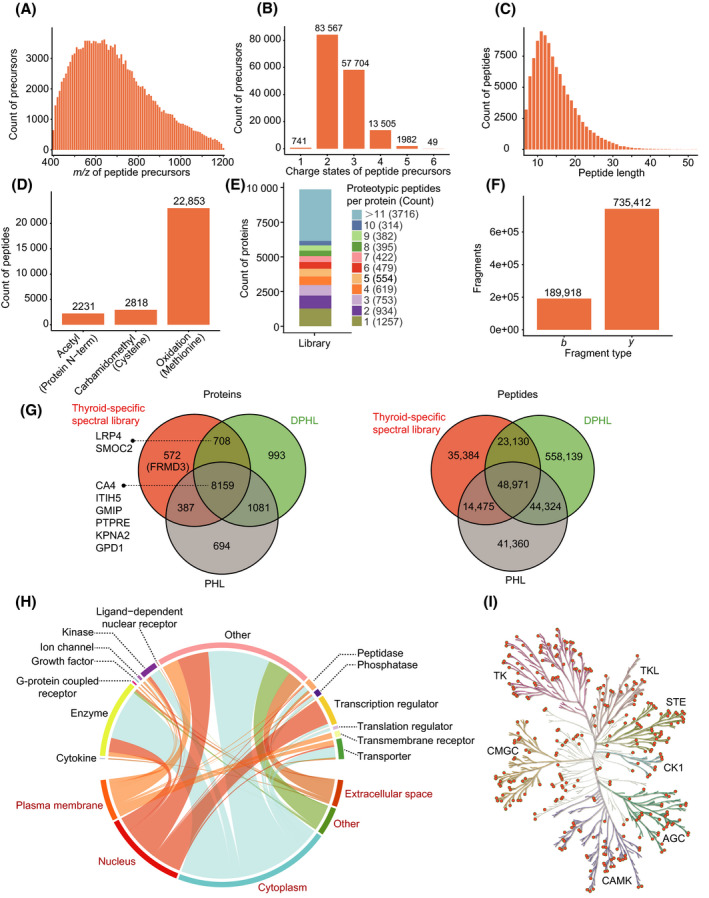
Characterization and statistics of the thyroid‐specific spectral library. (A) Distribution of peptide precursor *m/z*. (B) Counts of different precursor charge states. (C) Distribution of identified peptides lengths. (D) Modified peptides numbers and distribution of three modifications. (E) Numbers of proteotypic peptides for each protein and their corresponding ratios and counts. (F) Ion counts of each fragment type. (G) Venn diagrams of proteins and peptides in our thyroid‐specific spectral library, the PHL and DPHL libraries. (H) Proteins are annotated according to two classification systems, subcellular location (words in red) and function type (words in black). Each curve represents one protein, linking the protein function type with the corresponding subcellular location. (I) A total of 340 kinases (orange dots) belonging to seven families (highlighted by the different tree colours) are identified in our library.

In our spectral library, the peptide precursor *m/z* was between 400 Th and 1200 Th, and approximately 82% of the precursors were between *m/z* of 400‐850 Th (Fig. 2A). Precursors primarily displayed two (53%) or three (37%) charges, and their charge distributions were comparable to those of different spectral libraries (Fig. 2B) [27]. 82% peptides were 8–20 amino acids long, with a median length of 14 amino acids, consistent with the properties of trypsinized peptides (Fig. 2C). We next focused on peptide modifications. Oxidation on methionine, the most common modification in our library, was detected in 22 853 out of 121 960 peptides. Sample preparation generated 2818 carbamidomethyled peptides at cysteine residues and 2231 N‐terminal acetylated ones (Fig. 2D). A total of 7634 proteins were detected with at least three proteotypic peptides, and nearly half of the proteins were found with more than 10 peptides (Fig. 2E). Additionally, fragments from *y* ions were more frequently detected than those from *b* ions due to the collision mode (Fig. 2F). Compared with the existing spectral libraries, our thyroid‐specific spectral library includes 572 proteins and 35 384 peptides which are not covered in the published pan‐human spectral library, pan‐human spectral library (PHL) [14] and DIA pan‐human spectral library (DPHL) [15] (Fig. 2G).

We next used Gene Ontology (GO) to identify the main enriched protein categories within our library. A total of 9826 proteins were annotated by ipa software: the enriched protein cellular locations (red words) and protein functions (black words) are shown in Fig. 2H. By matching our data to the kinase database KinMap [26], our library was found to contain 340 kinases from seven families, accounting for 63.4% (340/536) of the entire kinase database (Fig. 2I). Thus, our library provides a valuable reference for applying the DIA‐MS method to human thyroid samples.

### 3.3 Technical validation on four datasets acquired by four strategies of DIA

**Fig. 3:**
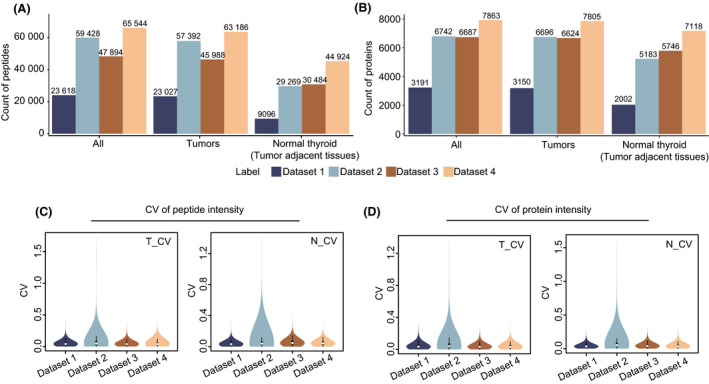
Results from a technical validation of our thyroid‐specific spectral library searched by Spectronaut. Four datasets were acquired with single‐shot DIA (dataset 1), PulseDIA (dataset 2), pre‐fractionation DIA (dataset 3) and a combination of pre‐fractionation and PulseDIA (dataset 4). Identified (A) peptides and (B) proteins were obtained by searching against our thyroid‐specific spectral library. Coefficient of variation of (C) peptides and (D) proteins abundance in tumours (T_CV) and their pericancer tissues (N_CV).

To further validate our library, we analysed three PTC samples, together with paired pericancer tissues. Four datasets were then acquired with the following four strategies: single‐shot DIA (dataset 1), PulseDIA (dataset 2), pre‐fractionation DIA (dataset 3), and a combination of pre‐fractionation and PulseDIA (dataset 4). All datasets were subsequently analysed using Spectronaut (version 14.6) and DIA‐NN (version 1.7.15) against our established thyroid tissue‐specific spectral library. The search results for the four datasets are shown in Fig. 3 and Fig. S1. All three tumour tissues expressed more proteins and peptides than the matched normal thyroid tissues (pericancer tissues), especially at the peptide level (Fig. 3A,B). The number of identified peptides and proteins using the single‐shot DIA were the fewest due to the relatively short gradient and the presence of a highly abundant protein, thyroglobulin. PulseDIA and pre‐fractionation DIA led to more identifications. PulseDIA identified more peptides than pre‐fractionation DIA, but a comparable number of proteins. The combination of pre‐fractionation and PulseDIA generated the best results at both peptide and protein levels: 65 544 peptides and 7863 proteins. DIA‐NN results (Fig. S1) were similar to the results searched by Spectronaut. These results showed that a longer gradient allows the detection of more peptides and proteins.

We next calculated the coefficient of variation (CV) of peptides and proteins abundance to evaluate the quality of these datasets. The median peptides CVs were less than 0.05 for all datasets (Fig. 3C). Similarly, the median protein CVs were all less than 0.04 (Fig. 3D). These results indicate that all four datasets performed well, as the quantitative differences were negligible. Thus, our spectral library is a valuable resource and provides a robust reference for proteomic exploration of thyroid disease.

### 3.4 Proteomic analysis of FA and FTC samples

**Fig. 4:**
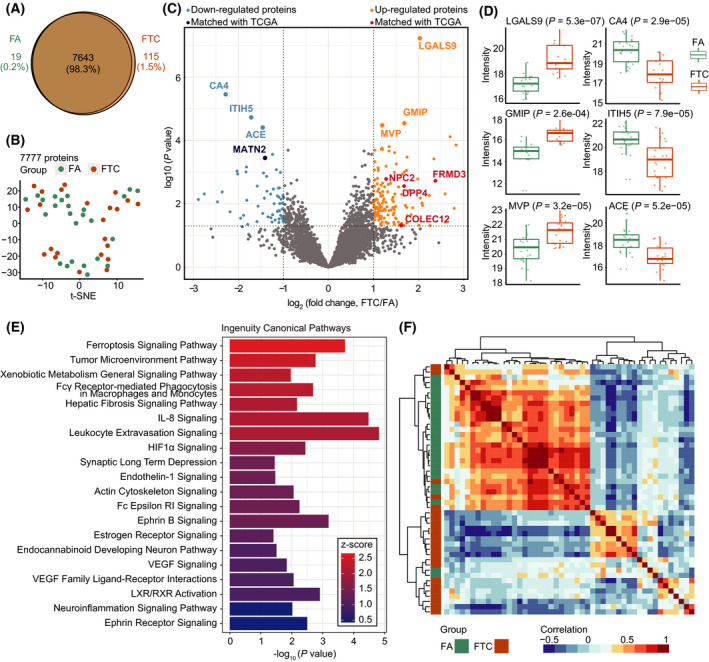
Stratification of follicular thyroid carcinoma and adenoma based on prototype analysed by DIA and the established spectral library. (A) Venn diagram shows 98.3% overlap of protein identification between FA and FTC. (B) The t‐SNE plot shows FA and FTC cannot be separated based on the expression of 7777 proteins. (C) Volcano plot highlights differentially expressed proteins with the threshold of |log_2_(fold change)| ≥ 1 and *P* value < 0.05. Red and orange points indicate upregulated proteins, while the light and dark blue points denote the downregulated proteins. Those six annotated points in light blue or orange are proteins with adjusted *P* values < 0.05. The red and dark blue points are overlapped with favourable/unfavourable prognosis genes of thyroid cancer acquired from TCGA transcriptomics data. (D) Protein expression plots for top six DEPs substantially dysregulated in FA and FTC. (E) Pathway enrichment analysis for 204 DEPs. Z‐score represents the degree of activation of the enriched pathway. (F) Unsupervised hierarchical clustering heatmap of Pearson correlation coefficients between every two samples on 31 proteins selected by geNetClassifier.

Next, we compared the proteomes of 46 follicular tumour samples for FA (*n* = 24) and FTC (*n* = 22), by PCT‐PulseDIA and DIA‐NN against the thus established thyroid spectral library. Two randomly selected samples from the 46 samples were treated as technical replicates and analysed twice. With the library, we identified 103 663 peptide precursors, 76 019 peptides and 7847 protein groups, and 7777 proteins. In all, 7643 of 7777 (98.3%) were identified in both FA and FTC (Fig. 4A). t‐Distributed Stochastic Neighbour Embedding (t‐SNE) analysis showed FA and FTC could not be distinguished by their global proteomes (Fig. 4B). Furthermore, a series of quality control analyses showed that we obtained high‐quality data with negligible artificial and biological bias (Fig. S2).

We next identified DEPs between the two sample types. There were 204 DEPs comprising 139 upregulated proteins, 56 downregulated proteins and 9 proteins that could be only detected in FTC (Fig. 4C). Five of 204 DEPs were matched with the list of 345 prognosis‐related genes of thyroid cancer analysed by The Cancer Genome Atlas (TCGA) transcriptome data. Next, we exhibited the expression of the top six substantially dysregulated proteins in box plots (Fig. 4D). Furthermore, we performed pathway enrichment analysis of the 204 DEPs. The most active pathway was the ferroptosis signaling pathway (Fig. 4E).

Moreover, we selected 31 proteins from the 204 DEPs to distinguish FTC and FA by geNetClassifier. Unsupervised clustering heatmap for Pearson correlation coefficients showed that FA and FTC were well separated based on the expression of the 31 proteins (Fig. 4F).

**Fig. 5:**
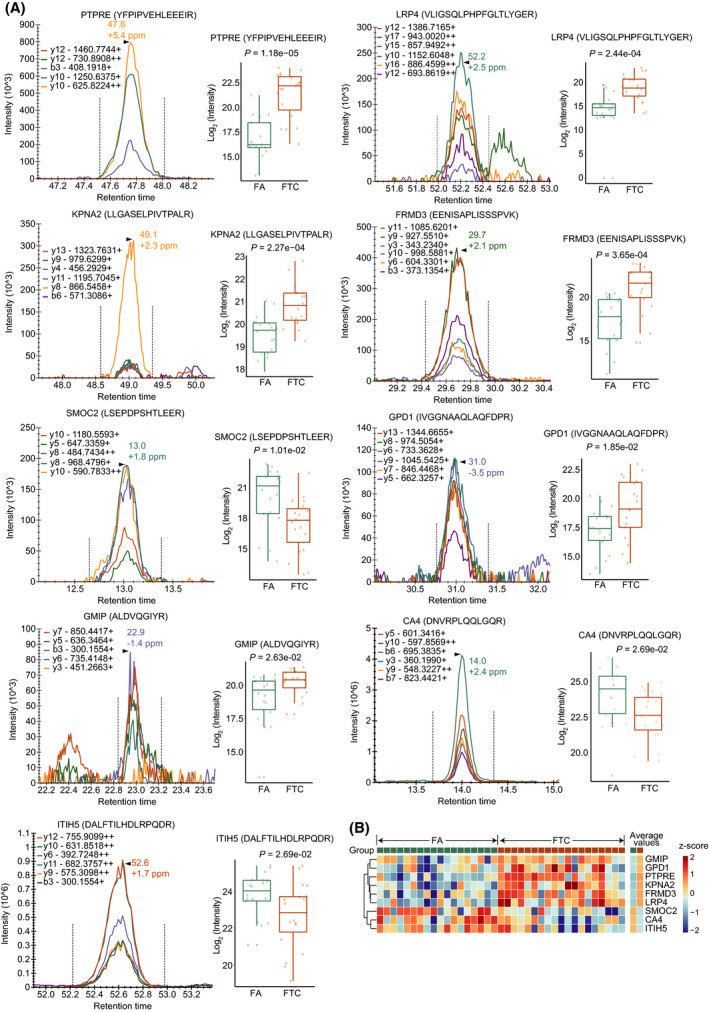
PRM‐MS analysis for nine selected proteins. (A) A representative peak group chromatography of a peptide precursor (left) and a box plot (right) showing the protein abundance in FA and FTC. Statistical significance was calculated by two‐tailed Student’s *t*‐test. (B) Heatmap showing the z‐score scaled expression of nine proteins in each sample, and the average expression of nine proteins in 18 FA and 19 FTC.

To further verify the reliability of differentially expressed and characteristic proteins from SVM, we collected another dataset from an independent hospital and detected the peptides from the selected proteins by PRM, a targeted proteomic strategy. From the 37 proteins (six proteins from DEPs and 31 proteins from geNetClassifier) detected by PRM, nine proteins were confirmed to be significantly dysregulated (adjusted *P* value < 0.05) in the new sample set, which was matched with the PulseDIA data as described above (Fig. 5A,B). Subcellular location and averaged expression in each group of the nine proteins are shown in Fig. S3A. Four of nine proteins are located in the membrane and four proteins are enzyme or phosphatase. The fold changes and adjusted *P* values of the two‐group comparison for the nine proteins are listed in Table 2.

**Table 2:** Nine differentially expressed proteins confirmed by PRM

Uniprot ID	Symbol	Entrez Gene Name	Subcellular location	log_2_(Fold change, FTC/FA)	Adjusted *P* value
P23469	PTPRE	Protein tyrosine phosphatase receptor type E	Plasma Membrane	1.2910	1.18E‐05
O75096	LRP4	LDL‐receptor‐related protein 4	Plasma Membrane	1.2679	2.44E‐04
P52292	KPNA2	Karyopherin subunit alpha 2	Nucleus	1.0668	2.27E‐04
A2A2Y4	FRMD3	FERM domain containing 3	Plasma Membrane	1.2227	3.65E‐04
Q9H3U7	SMOC2	SPARC‐related modular calcium binding 2	Extracellular Space	0.8629	1.01E‐02
P21695	GPD1	Glycerol‐3‐phosphate dehydrogenase 1	Cytoplasm	1.1141	1.85E‐02
Q9P107	GMIP	GEM interacting protein	Cytoplasm	1.0664	2.63E‐02
P22748	CA4	Carbonic anhydrase 4	Plasma Membrane	0.9398	2.69E‐02
Q86UX2	ITIH5	Inter‐alpha‐trypsin inhibitor heavy chain 5	Extracellular Space	0.9516	2.69E‐02

To validate the performance of the nine characteristic proteins acquired by PRM on distinguishing FA and FTC, we trained a SVM classifier using the same hyper‐parameters setting as previously employed for the 31 proteins of PulseDIA data. The model achieved an AUC of 0.963 and an accuracy of 91.7% on the testing set (Fig. S3B). Furthermore, FA and FTC samples were satisfactorily separated in the t‐SNE plot based on the nine proteins (Fig. S3C). Our data suggest that these nine proteins are promising biomarkers for distinguishing FA and FTC tumours.

## 4 Discussion

Differential diagnosis of benign and malignant follicular thyroid tumours, that is, FA and FTC, remains a great challenge. Histologically, the benign and malignant tumour cells are identical under microscopy, and no genomic aberrancy has been identified to distinguish them. In this study, we analysed the proteome of FA and FTC with a goal to explore whether there are differentially expressed proteins that can be used to separate them. The proteome of thyroid tumours has been understudied compared to many other common tumours. In the literature, up to 2682 proteins have been characterized for thyroid tumours [28]. Here, we present a thyroid‐specific spectral library that comprises 925 330 transition groups, 157 548 precursors, 121 960 peptides, 9941 protein groups and 9826 proteins from proteotypic peptides, which largely expands our understanding of proteins expressed in thyroid tumours. Compared with the existing pan‐human spectral libraries, our thyroid‐specific spectral library includes 572 proteins and 35 384 peptides which are not covered in those libraries [14, 15] (Fig. 2G). This thyroid‐specific spectral library offers a specific and in‐depth spectral resource for studying the proteome of thyroid tissues, allowing exploration of potential proteins differentiating FA and FTC.

Then we analysed the proteome of 24 FA and 22 FTC tumours using PulseDIA and this spectral library, leading to characterization of 7777 proteins, in which 204 DEPs were identified. Of these, three proteins were reported as relevant for FTC, namely, carbonic anhydrase 4 (CA4) [29], inter‐alpha‐trypsin inhibitor heavy chain H5 (ITIH5) [30] and angiotensin‐converting enzyme (ACE) [31]. Three proteins have not previously been associated with FTC, but are related to thyroid function or other types of cancer, namely, major vault protein (MVP) [32], GEM‐interacting protein (GMIP) [33] and galectin‐9 (LGALS9) [34]. Furthermore, the pathway enrichment analysis of the 204 DEPs shows that the ferroptosis signaling pathway is the most active pathway (Fig. 4E), which has not been associated with FTC but reported in other types of cancers [35]. Ferroptosis was first proposed as a novel mechanism of cell death, which suggests a perspective in cancer therapeutics. Ferroptosis is associated with the tumour immune microenvironment and other enriched pathways, such as tumour microenvironment pathway, leukocyte extravasation signaling and IL‐8 signaling [36].

Next, we prioritized 31 proteins to distinguish FA and FTC using geNetClassifier. Together with top six DEPs, we employed PRM to check the expression of these 37 protein candidates in an independent sample set, containing 18 FA and 19 FTC samples, from a different hospital. Nine proteins were confirmed in this independent sample set. Three of the nine proteins have been involved in FTC. Higher expression of SPARC‐related modular calcium binding 2 (SMOC2), a secreted protein, in FA than in FTC has been observed using tissue microarrays (TMA) [37]. Moreover, the high mRNA expression of SMOC2 was associated with better outcomes in patients with PTC [37]. ITIH5 is a member of inter‐alpha‐trypsin inhibitor (ITI) family, which functions as a tumour suppressor in breast cancer [38] and thyroid cancer [39]. Furthermore, Pfeifer et al. built up a five‐gene‐based classifier based on mRNA expression to distinguish FA and FTC, and ITIH5 was a member of the panel [30]. Downregulated expression of CA4 in FTC was reported in a sample set of 26 FTC and 53 FA [29], in consistent with our observation here. Two of the nine proteins were reported to be modulated in PTC, namely, LDL‐receptor‐related protein 4 (LRP4) and protein tyrosine phosphatase receptor type E (PTPRE). LRP4 was found significantly upregulated in PTC compared with normal thyroid tissues by gene chip and validated by quantitative polymerase chain reaction (qPCR) [40]. The mRNA level of LRP4 has been utilized to construct a panel to distinguish PTC from normal thyroid tissue [40]. PTPRE was upregulated in PTC compared with normal thyroid tissue based on TCGA dataset [41]. The remaining four proteins have not been reported in thyroid cancer, awaiting further investigation.

## 5 Conclusion

In conclusion, we present a comprehensive spectral library for DIA and PRM analysis of thyroid tissue samples and identified nine characteristic proteins that can be used to separate FA and FTC. Further validation of these potential biomarkers in independent cohorts is required in future studies.

## Author contributions

TG and YS designed the project. NGI and OLK provided Singapore set and YZ, XT, GW, YH and JX collected the Chinese sets. YS, LL, WL, QX and XC performed the experiments. YS, LL, YZ, WG, HW, RW and HC conducted the data analysis. YS and TG wrote the manuscript with inputs from all co‐authors. TG, YZ, YZ and YS supervised the project.

## Conflict of interest

YZ and TG are shareholders of Westlake Omics Inc. WG, WL, HW and HC are employees of Westlake Omics Inc. The other authors declare no competing interests in this paper.

### Peer review

The peer review history for this article is available at https://publons.com/publon/10.1002/1878‐0261.13198.

## Supporting information


**Fig. S1**. Results from a technical validation of our thyroid‐specific spectral library analyzed by DIA‐NN.

**Fig. S2**. Proteomic data quality control.

**Fig. S3**. Subcellular location of the nine proteins and performance of the nine‐protein classifier.

**Table S1**. Patient information.

**Table S2**. Data‐dependent acquisition information.

## Data Availability

The mass spectrometry proteomics data have been deposited to the ProteomeXchange Consortium via the PRIDE [42] partner repository with the dataset identifier PXD026395.

## References

[1] Burman KD , Wartofsky L . Thyroid nodules. N Engl J Med. 2015;373:2347–56. 10.1056/NEJMcp1415786. 26650154

[2] Singh Ospina N , Iniguez‐Ariza NM , Castro MR . Thyroid nodules: diagnostic evaluation based on thyroid cancer risk assessment. BMJ. 2020;368:l6670. 10.1136/bmj.l6670. 31911452

[3] Lim H , Devesa SS , Sosa JA , Check D , Kitahara CM . Trends in thyroid cancer incidence and mortality in the United States, 1974–2013. JAMA. 2017;317:1338–48. 10.1001/jama.2017.2719. 28362912 PMC8216772

[4] Miranda‐Filho A , Lortet‐Tieulent J , Bray F , Cao B , Franceschi S , Vaccarella S , et al. Thyroid cancer incidence trends by histology in 25 countries: a population‐based study. Lancet Diabetes Endocrinol. 2021;9:225–34. 10.1016/S2213-8587(21)00027-9. 33662333

[5] Fagin JA , Wells SA Jr . Biologic and clinical perspectives on thyroid cancer. N Engl J Med. 2016;375:1054–67. 10.1056/NEJMra1501993. 27626519 PMC5512163

[6] Alexander EK , Kennedy GC , Baloch ZW , Cibas ES , Chudova D , Diggans J , et al. Preoperative diagnosis of benign thyroid nodules with indeterminate cytology. N Engl J Med. 2012;367:705–15. 10.1056/NEJMoa1203208. 22731672

[7] Daniels GH . Follicular thyroid carcinoma: a perspective. Thyroid. 2018;28:1229–42. 10.1089/thy.2018.0306. 30039751

[8] Wang TS , Sosa JA . Thyroid surgery for differentiated thyroid cancer ‐ recent advances and future directions. Nat Rev Endocrinol. 2018;14:670–83. 10.1038/s41574-018-0080-7. 30131586

[9] Gillet LC , Navarro P , Tate S , Rost H , Selevsek N , Reiter L , et al. Targeted data extraction of the MS/MS spectra generated by data‐independent acquisition: a new concept for consistent and accurate proteome analysis. Mol Cell Proteomics. 2012;11:111. 10.1074/mcp.O111.016717. PMC3433915 22261725

[10] Zhang F , Ge W , Ruan G , Cai X , Guo T . Data‐independent acquisition mass spectrometry‐based proteomics and software tools: a glimpse in 2020. Proteomics. 2020;20:e1900276. 10.1002/pmic.201900276. 32275110

[11] Gao H , Zhang F , Liang S , Zhang Q , Lyu M , Qian L , et al. Accelerated lysis and proteolytic digestion of biopsy‐level fresh‐frozen and FFPE tissue samples using pressure cycling technology. J Proteome Res. 2020;19:1982–90. 10.1021/acs.jproteome.9b00790. 32182071

[12] Guo T , Kouvonen P , Koh CC , Gillet LC , Wolski WE , Rost HL , et al. Rapid mass spectrometric conversion of tissue biopsy samples into permanent quantitative digital proteome maps. Nat Med. 2015;21:407–13. 10.1038/nm.3807. 25730263 PMC4390165

[13] Schubert OT , Gillet LC , Collins BC , Navarro P , Rosenberger G , Wolski WE , et al. Building high‐quality assay libraries for targeted analysis of SWATH MS data. Nat Protoc. 2015;10:426–41. 10.1038/nprot.2015.015. 25675208

[14] Rosenberger G , Koh CC , Guo T , Rost HL , Kouvonen P , Collins BC , et al. A repository of assays to quantify 10,000 human proteins by SWATH‐MS. Sci Data. 2014;1:140031. 10.1038/sdata.2014.31. 25977788 PMC4322573

[15] Zhu T , Zhu Y , Xuan Y , Gao H , Cai X , Piersma SR , et al. DPHL: A DIA Pan‐human Protein Mass Spectrometry Library for Robust Biomarker Discovery. Genom Proteom Bioinform. 2020;18:104–19. 10.1016/j.gpb.2019.11.008. PMC7646093 32795611

[16] Ge W , Liang X , Zhang F , Hu Y , Xu L , Xiang N , et al. Computational optimization of spectral library size improves DIA‐MS proteome coverage and applications to 15 tumors. J Proteome Res. 2021;20:5392–401. 10.1021/acs.jproteome.1c00640. 34748352

[17] Tsou CC , Avtonomov D , Larsen B , Tucholska M , Choi H , Gingras AC , et al. DIA‐Umpire: comprehensive computational framework for data‐independent acquisition proteomics. Nat Methods. 2015;12:258–64. 10.1038/nmeth.3255. 25599550 PMC4399776

[18] Ting YS , Egertson JD , Bollinger JG , Searle BC , Payne SH , Noble WS , et al. PECAN: library‐free peptide detection for data‐independent acquisition tandem mass spectrometry data. Nat Methods. 2017;14:903–8. 10.1038/nmeth.4390. 28783153 PMC5578911

[19] Demichev V , Messner CB , Vernardis SI , Lilley KS , Ralser M . DIA‐NN: neural networks and interference correction enable deep proteome coverage in high throughput. Nat Methods. 2020;17:41–4. 10.1038/s41592-019-0638-x. 31768060 PMC6949130

[20] Blattmann P , Stutz V , Lizzo G , Richard J , Gut P , Aebersold R . Generation of a zebrafish SWATH‐MS spectral library to quantify 10 000 proteins. Sci Data. 2019;6:190011. 10.1038/sdata.2019.11. 30747917 PMC6371892

[21] Sun Y , Selvarajan S , Zang Z , Liu W , Zhu Y , Zhang H , et al. Protein classifier for thyroid nodules learned from rapidly acquired proteotypes. medRxiv. 2020 [PREPRINT].

[22] Zhu Y , Weiss T , Zhang Q , Sun R , Wang B , Yi X , et al. High‐throughput proteomic analysis of FFPE tissue samples facilitates tumor stratification. Mol Oncol. 2019;13:2305–28. 10.1002/1878-0261.12570. 31495056 PMC6822243

[23] Cai X , Ge W , Yi X , Sun R , Zhu J , Lu C , et al. PulseDIA: data‐independent acquisition mass spectrometry using multi‐injection pulsed gas‐phase fractionation. J Proteome Res. 2020;20:279–88. 10.1021/acs.jproteome.0c00381. 32975123

[24] Aibar S , Fontanillo C , Droste C , Roson‐Burgo B , Campos‐Laborie FJ , Hernandez‐Rivas JM , et al. Analyse multiple disease subtypes and build associated gene networks using genome‐wide expression profiles. BMC Genom. 2015;16(Suppl 5):S3. 10.1186/1471-2164-16-S5-S3. PMC4460584 26040557

[25] Lang M , Binder M , Richter J , Schratz P , Pfisterer F , Coors S , et al. mlr3: A modern object‐oriented machine learning framework in R. J Open Source Software. 2019;4:1903. 10.21105/joss.01903.

[26] Eid S , Turk S , Volkamer A , Rippmann F , Fulle S . KinMap: a web‐based tool for interactive navigation through human kinome data. BMC Bioinform. 2017;18:16. 10.1186/s12859-016-1433-7. PMC5217312 28056780

[27] Zhang H , Liu P , Guo T , Zhao H , Bensaddek D , Aebersold R , et al. Arabidopsis proteome and the mass spectral assay library. Sci Data. 2019;6:278. 10.1038/s41597-019-0294-0. 31757973 PMC6874543

[28] Martínez‐Aguilar J , Clifton‐Bligh R , Molloy MP . Proteomics of thyroid tumours provides new insights into their molecular composition and changes associated with malignancy. Sci Rep. 2016;6:23660. 10.1038/srep23660. 27025787 PMC4812243

[29] Davidov T , Nagar M , Kierson M , Chekmareva M , Chen C , Lu SE , et al. Carbonic anhydrase 4 and crystallin alpha‐B immunoreactivity may distinguish benign from malignant thyroid nodules in patients with indeterminate thyroid cytology. J Surg Res. 2014;190:565–74. 10.1016/j.jss.2014.03.042. 24880201 PMC5767464

[30] Pfeifer A , Wojtas B , Oczko‐Wojciechowska M , Kukulska A , Czarniecka A , Eszlinger M , et al. Molecular differential diagnosis of follicular thyroid carcinoma and adenoma based on gene expression profiling by using formalin‐fixed paraffin‐embedded tissues. BMC Med Genomics. 2013;6:38. 10.1186/1755-8794-6-38. 24099521 PMC3852913

[31] Narayan SS , Lorenz K , Ukkat J , Hoang‐Vu C , Trojanowicz B . Angiotensin converting enzymes ACE and ACE2 in thyroid cancer progression. Neoplasma. 2020;67:402–9. 10.4149/neo_2019_190506N405. 31847529

[32] Teng Y , Ren Y , Hu X , Mu J , Samykutty A , Zhuang X , et al. MVP‐mediated exosomal sorting of miR‐193a promotes colon cancer progression. Nat Commun. 2017;8:14448. 10.1038/ncomms14448. 28211508 PMC5321731

[33] Colas E , Perez C , Cabrera S , Pedrola N , Monge M , Castellvi J , et al. Molecular markers of endometrial carcinoma detected in uterine aspirates. Int J Cancer. 2011;129:2435–44. 10.1002/ijc.25901. 21207424

[34] Luo LH , Li DM , Wang YL , Wang K , Gao LX , Li S , et al. Tim3/galectin‐9 alleviates the inflammation of TAO patients via suppressing Akt/NF‐kB signaling pathway. Biochem Biophys Res Commun. 2017;491:966–72. 10.1016/j.bbrc.2017.07.144. 28756232

[35] Mou Y , Wang J , Wu J , He D , Zhang C , Duan C , et al. Ferroptosis, a new form of cell death: opportunities and challenges in cancer. J Hematol Oncol. 2019;12:34. 10.1186/s13045-019-0720-y. 30925886 PMC6441206

[36] Ge M , Niu J , Hu P , Tong A , Dai Y , Xu F , et al. A Ferroptosis‐related signature robustly predicts clinical outcomes and associates with immune microenvironment for thyroid cancer. Front Med (Lausanne). 2021;8:637743. 10.3389/fmed.2021.637743. 33928101 PMC8076739

[37] Kim HS , Choi JH , Lee JY , Kang J , Myung JK , Kim WH , et al. Downregulation of SMOC2 expression in papillary thyroid carcinoma and its prognostic significance. Sci Rep. 2020;10:4853. 10.1038/s41598-020-61828-z. 32184420 PMC7078233

[38] Veeck J , Chorovicer M , Naami A , Breuer E , Zafrakas M , Bektas N , et al. The extracellular matrix protein ITIH5 is a novel prognostic marker in invasive node‐negative breast cancer and its aberrant expression is caused by promoter hypermethylation. Oncogene. 2008;27:865–76. 10.1038/sj.onc.1210669. 17653090

[39] Pita JM , Banito A , Cavaco BM , Leite V . Gene expression profiling associated with the progression to poorly differentiated thyroid carcinomas. Br J Cancer. 2009;101:1782–91. 10.1038/sj.bjc.6605340. 19809427 PMC2778548

[40] Jarzab B , Wiench M , Fujarewicz K , Simek K , Jarzab M , Oczko‐Wojciechowska M , et al. Gene expression profile of papillary thyroid cancer: sources of variability and diagnostic implications. Cancer Res. 2005;65:1587–97. 10.1158/0008-5472.CAN-04-3078. 15735049

[41] Yang F , Zhang J , Li B , Zhao Z , Liu Y , Zhao Z , et al. Identification of Potential lncRNAs and miRNAs as diagnostic biomarkers for papillary thyroid carcinoma based on machine learning. Int J Endocrinol. 2021;2021:3984463. 10.1155/2021/3984463. 34335744 PMC8318749

[42] Perez‐Riverol Y , Csordas A , Bai J , Bernal‐Llinares M , Hewapathirana S , Kundu DJ , et al. The PRIDE database and related tools and resources in 2019: improving support for quantification data. Nucleic Acids Res. 2019;47:D442–50. 10.1093/nar/gky1106. 30395289 PMC6323896

