# Supplementary figures and images for: Stratification of follicular thyroid tumours using data‐independent acquisition proteomics and a comprehensive thyroid tissue spectral library
Source: Mol Oncol. 2022 Mar 12;16(8):1611–24. doi: 10.1002/1878-0261.13198 (PMC9019893; doi:10.1002/1878-0261.13198)

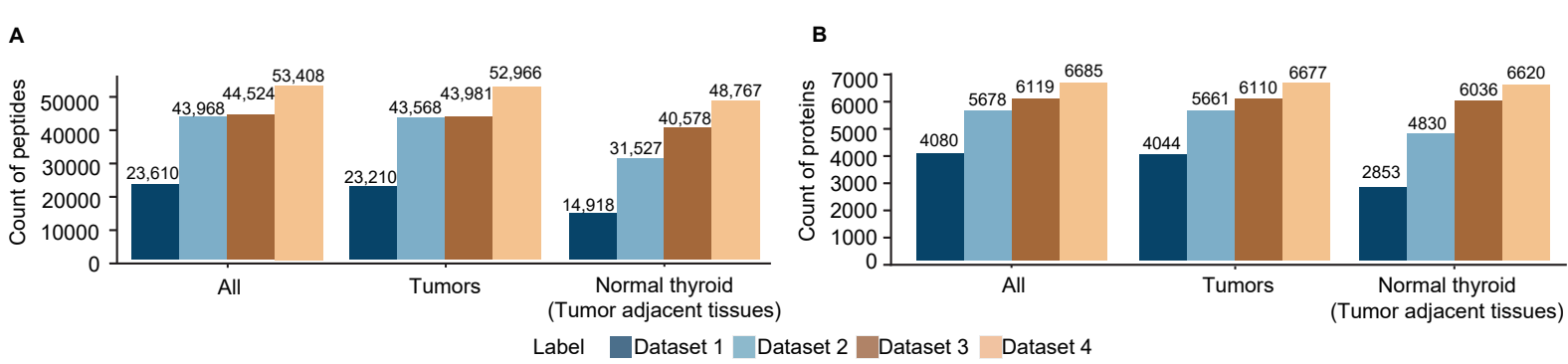

Supplement: Supplementary file 1 — Fig. S1. Results from a technical validation of our thyroid‐specific spectral library analyzed by DIA‐NN. [file MOL2-16-1611-s004.pdf]

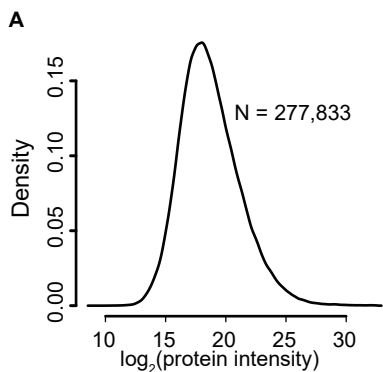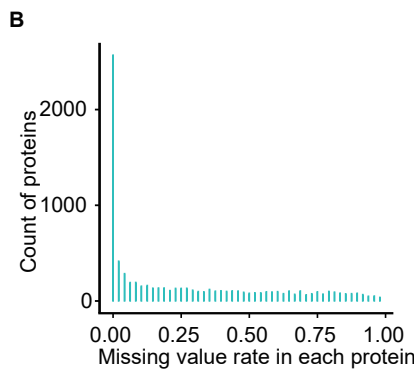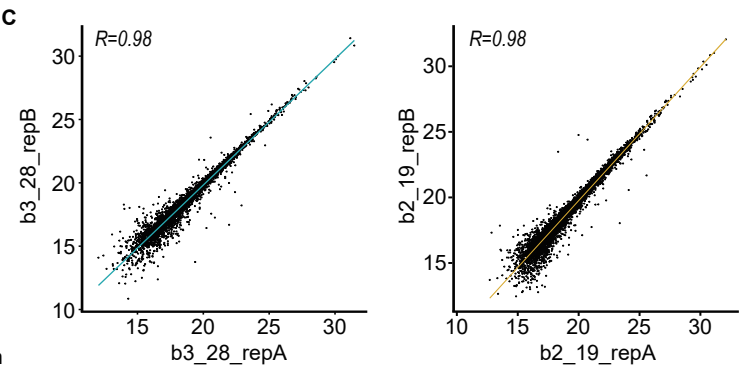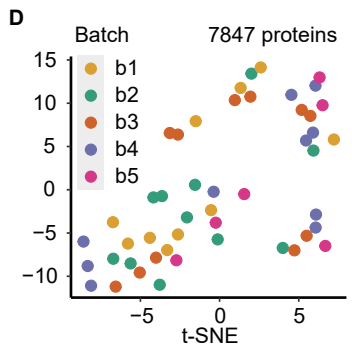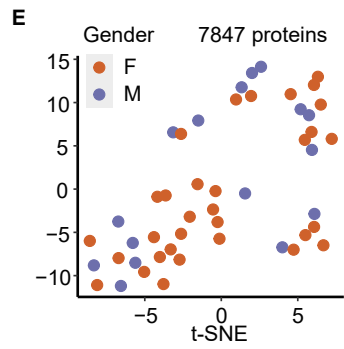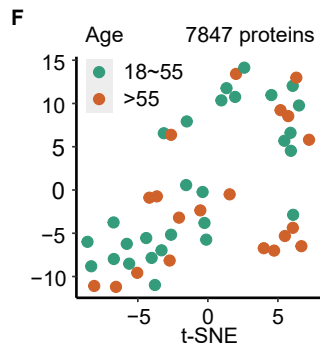

Supplement: Supplementary file 2 — Fig. S2. Proteomic data quality control. [file MOL2-16-1611-s002.pdf]

A

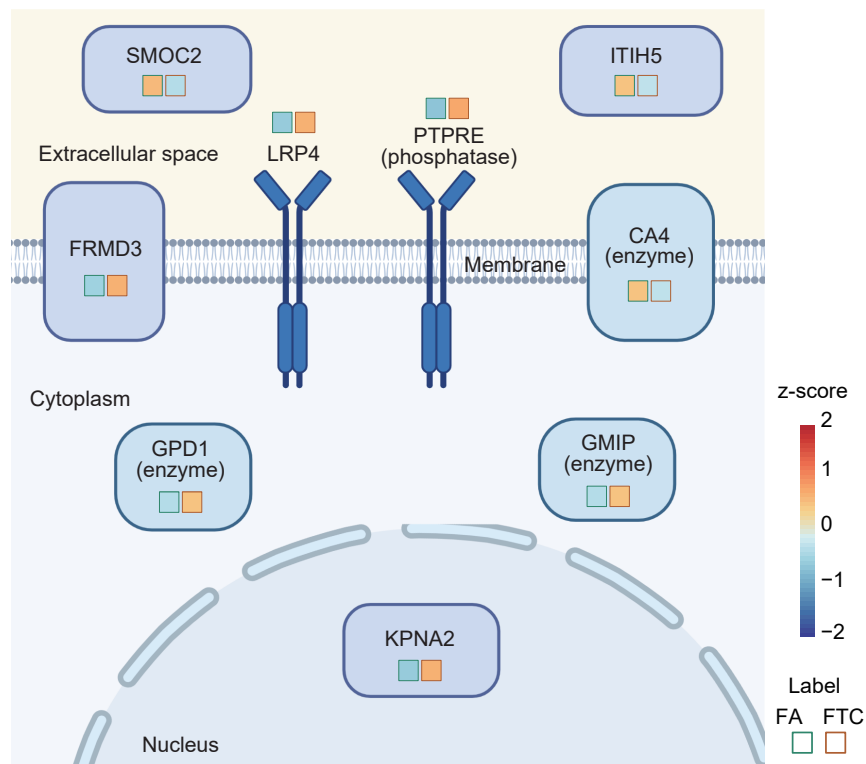

B

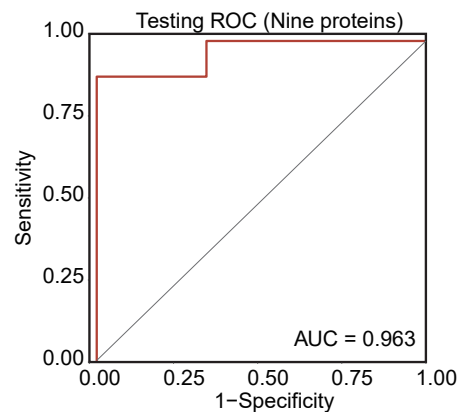

C

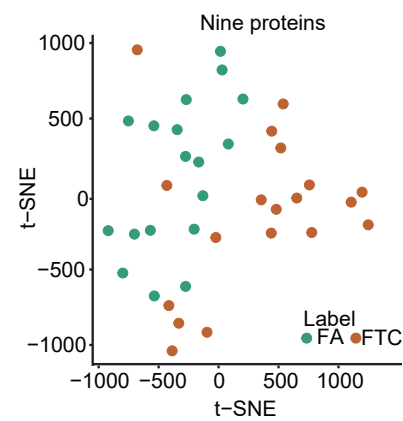

Supplement: Supplementary file 3 — Fig. S3. Subcellular location of the nine proteins and performance of the nine‐protein classifier. [file MOL2-16-1611-s003.pdf]
